# Affective associations towards running: fuzzy patterns of implicit-explicit interaction in young female runners and non-runners

**DOI:** 10.3389/fspor.2024.1210546

**Published:** 2024-01-31

**Authors:** Tim Burberg, Sabine Würth, Günter Amesberger, Thomas Finkenzeller

**Affiliations:** Department of Sport and Exercise Science, University of Salzburg, Salzburg, Austria

**Keywords:** affective associations, fuzzy cluster analysis, implicit-explicit interaction, running behavior, young women

## Abstract

Empirical evidence demonstrates that high concordance and low discrepancy of implicit and explicit affective processes facilitate consistent exercise behavior. Novice runners often have difficulties implementing their running behavior on a regular basis resulting in irregular running behavior. To investigate the potential value of affective associations 89 young female runners (regular and irregular) and non-runners were recruited. Affective associations towards running were measured through a Single-Target Implicit Association Test on the implicit level and by self-report on the explicit level. Implicit-explicit interaction (IEI) scores (i.e., implicit-explicit concordance and discrepancy) were derived from principal component analysis. Fuzzy k-means cluster analysis was used to identify patterns of interacting implicit-explicit affective associations. The resulting clusters were assessed for differences in previous running experience, current running behavior, motivational and intentional aspects. Four meaningful overlapping clusters were found and labeled according to their prevalent IEI patterns (i.e., “positive non-discrepant”, “positive discrepant”, “negative discrepant”, “negative non-discrepant”). Significant differences between clusters were found for past running experience, current running behavior, motivational and intentional aspects. The results indicate that running behavior varies between and within patterns of affective associations. In line with previous findings, positive non-discrepant implicit and explicit affective associations are linked to more consistent running behavior, while negative non-discrepant affect is associated with non-runners. However, the occurrence of discrepant implicit-explicit affective associations in young women differing in running behavior, motivation, and intention broadens the view of the complex relationship between affective processes and exercise behavior. In conclusion, individualized interventions that take into account the implicit-explicit interaction of affective associations besides well-known cognitive self-regulatory resources may prove more effective for individuals who struggle to run regularly.

## 1 Introduction

Maintaining a regular running habit has the potential to provide various health benefits that can counter public health issues (1). These issues are partly caused by steadily increasing levels of physical inactivity (2). Running is simple in its performance and relatively easy to integrate in everyday life. Yet, people often struggle to put their intentions into action. A phenomenon which is referred to as the intention-behavior gap (IBG) and quite prevalent for physical activities like running (3, 4). For instance, approximately 72% of novice runners who discontinued running after a running program had previously expressed their intention to continue running after completing the program. Further, approximately 78% stated in the follow-up survey that they intend to start running again in the future (5). In addition, mainly women were affected by the IBG in this study (81.8%). Due to the elevated incidence of physical inactivity especially among women in European nations compared to men (2), mechanisms linked to the IBG in running-related activities among women should be further investigated.

An important key factor to bridge the IBG is perceived positive affect (such as enjoyment and pleasure) during exercise (6, 7). In the context of running, short- and long-term increases in positive affect during running were associated with increased follow-up exercise behavior (8, 9). Besides, several authors found that positive affect towards running was positively associated with higher participation rates in running events and running behavior (10–15). Further, positive affect towards running was found to coincide with higher running experience and frequency as more experienced runners (i.e., more than eight years) emphasize that they are “running for the love of it” (16). In contrast, negative affect (e.g., feeling demotivated/tired, running is not the preferred sport) and situational barriers (e.g., weather, no time) were associated with poor adherence or discontinuation of running (5, 17). Thus, experienced runners appear to have accumulated resources to bridge the IBG that are in part associated with positive affective processes that less experienced runners lack. Furthermore, the potential benefits of positive affect are particularly evident for female runners when considering their perception of affective experiences. Research from neuroscience suggests that women are vigilant for emotional stimuli and recall of past experiences is enhanced by affective associations (18). Thus, the valence of the running experience and the resulting affective association may be especially relevant in how women perceive running. There is a lack of empirical studies looking specifically at positive affect towards running in women. Instead, concepts women deem important in regard with running (15, 19, 20) that are closely linked to positive affect — like affiliation, improving psychological coping and self-esteem (21–23) — provide valuable indirect evidence. For example, a bi-directional link between self-esteem and running behavior was found, as women who engage in regular running behavior experience an improvement in self-esteem, which in turn increases their likelihood of maintaining their running behavior (24). Additionally, repeated running sessions might be experienced more positive over time (e.g., adaptation of breathing, feeling more vital) leading to more regular running behavior.

Integrating affective processes is a relatively new development in the field of exercise psychological research. Historically, research in exercise psychology has primarily focused on explicit cognitive processes to explain exercise behavior. Therefore, most interventions to change exercise behavior are designed accordingly by targeting participants’ reflective abilities. Accumulating research indicates that explicit processes are insufficient in their prediction of exercise and physical activity behavior. Therefore, some authors argue that interventions based solely on these findings do not adequately address the IBG (25–28). Emphasizing the importance of affect and adding the perspective of implicit processes, several so called dual-process theories have emerged in the area of exercise psychology (29–31). In general, implicit processes are described as fast and automatic in their activation and mostly unavailable to consciousness, while explicit processes refer to slower conscious reflections towards the target behavior (e.g., running) (32, 33). Empirical data suggest that implicit and explicit processes explain unique portions of behavioral variance towards exercise and physical activity behavior and hence, are partially independent from each other (34–36). However, meta-analytic research indicates that the independent contribution of implicit processes is rather small (*r* = 0.10) (37). Yet, the interaction of implicit and explicit processes and their role in bridging the IBG is gaining attention (38–42).

Several theoretical perspectives propose that implicit and explicit processes are interrelated, and their interaction (IEI) is assumed to be critical for subsequent behavioral decision-making (43–46). More specifically, the degree of discrepancy between implicit and explicit processes might reflect ambivalent affective experiences with the target behavior. Discrepant implicit and explicit associations are assumed to potentially hinder goal achievement (i.e., translating intentions into action) by having a detrimental effect on self-regulatory processes (29, 47). Implicit-explicit discrepancy (IED) was found to be linked to higher dropout rates in an exercise program, a higher mismatch between desired and actual exercise behavior, lower physical activity levels, and a reduced likelihood of successfully adopting physical activity behavior (38–42). In contrast, greater implicit-explicit concordance (IEC) (i.e., combined positive valence of implicit and explicit associations) was associated with higher objective exercise frequency and higher adherence to an exercise program (38, 39). Until now, this relationship was primarily studied in regard with physical activity in general (38–42). However, individuals can hold different affective associations towards different physical activities (10, 11, 48, 49). Investigating implicit-explicit interaction of affective associations in a specific domain like running in women may provide a more differentiated view on this field of research. In addition, more accurate inferences can be derived for a specific form of exercise and target group. Looking at affective processes in young women varying in running behavior (i.e., regular runners, irregular runners and non-runners) might help to understand the potential value of these processes for individuals who have difficulties to run regularly.

The aim of this study was to identify patterns of implicit and explicit affective associations that women relate with running and how these are interlinked to running behavior and motivational aspects. First, implicit and explicit affective associations towards running and their interaction (i.e., IEC and IED) were calculated and described. Second, patterns of IEI were explored using cluster analysis. Then, the resulting clusters of IEI patterns were compared in regard with running behavior. We hypothesize that affective associations are formed by past experience, reflect current behavior and are relevant for future behavioral decision-making. Hence, running behavior was assessed taking into account past, present and future time perspectives. Thus, conclusions on how distinct patterns of IEI are related to running behavior of young female runners and non-runners can be drawn.

## 2 Methods

### 2.1 Participants

A sample of 89 young female adults between 18 and 30 years (*M* = 23.4 years, *SD* = 3.4) participated in the study. 29 participants (32.6%) reported having a professional job, while 56 participants (62.9%) were currently enrolled in a university program. Among the university students, four also held a professional job in addition to their studies. Two participants reported going to high school at the time of data collection. Also, two participants did not provide occupational information. 77 participants (86.5%) were identified as being right-handed, six participants (6.7%) as left-handed and another six as ambidextrous (6.7%) (see Table 1). Handedness was assessed by a modified 8-item version of the Edinburgh Handedness Inventory (EHI) (50). Items “Writing”, “Throwing”, “Scissors”, “Toothbrush”, “Knife”, “Spoon” and “Striking match” from the original inventory were used in addition to the item ‘Computer Mouse’. The scale range included the response options “Always Left”, “Usually Left”, “No Preference”, “Usually Right” and “Always Right”. Respondents were classified as left-handed (−100 to −50), ambidextrous (−49 to +49) or right-handed (+50 to +100). Characteristics of the participants’ running and exercise behavior are part of the outcome variables of this article and are therefore described in the results section (see 3.2 and 3.3).

**Table 1:** Participant characteristics (*n* = 89).

	*n*	*M*	*SD*	Range
Age [years]	89	23.4	3.4	18–30
Profession				
High school student	2			
University student	56			
Working	33^a^			
Handedness				
Right-handed	77			
Ambidextrous	6			
Left-handed	6			

^a^ Includes working individuals and university students pursuing a professional job in addition to their studies.

### 2.2 Procedure

Data collection was carried out online using the QDesigner Software (©Amescon). The QDesigner software facilitates collection of questionnaire and objective performance data. Participants were recruited via e-mail, social media and messenger applications. Task instructions and a link to the questionnaires and ST-IAT were provided via e-mail. Participants were instructed to execute the study in a quiet environment. Data collection was conducted in the following order: informed consent, demographics, socioeconomics, handedness, implicit measure, explicit self-report measures. The whole procedure took approximately 20 minutes. Stimuli material and the German versions of the self-report measures can be found in the Supplementary Materials 1, 2, respectively. An overview about definitions and scoring of variables used for analysis are provided in Table 2.

**Table 2:** Definitions and scoring information about variables of affective associations, running experience, running behavior, intention and motivation.

Variable	Definition	Instrument	Scoring
Affective associations			
Implicit	Fast and automatic activation of past experiences stored as affective associations in memory. These processes are not necessarily available to consciousness and are therefore implicitly (indirectly) accessed (33, 58).	ST-IAT (51)	Implicit affective associations were calculated as mean standardized rank difference between association conditions including practice and critical trials (G-Score) (68).
Explicit	Result of slow(er) cognitive processes of reflectively evaluating a target object/behavior, which is—in this case—affectively laden (33).	Feeling Thermometer (66)	Single item scale (11-point Likert scale)
Implicit-explicit interaction	Degree of agreement between implicit and explicit association operationalized as combined valence (i.e., IEC) and directed difference (i.e., IED). This interactive relationship represents the valence and extent of conflicting affective processes towards a target object/behavior (43–46).	PCA Scores (39)	Two components representing IEC and IED were extracted from implicit and explicit association values using principal component analysis.
Past perspective on running intention & behavior			
Past regular running experience	Previous experience with regular running and current intention to start running regularly (again).	–	Categorization as “No intention & no experience”, “No intention & past experience”, “Intention & no experience” “Intention & past experience”, “Regular runner”
Present perspective on running intention & behavior			
Current running behavior	Current running behavior was assessed in terms of regularity in retrospect of the past six months.	–	Categorization as regular, irregular runner or non-runner.
Motivation	Current running-related motivation was assessed as self-concordance and its subdimensions of intrinsic, identified, introjected and extrinsic motivation.	SSK-scale (71)	Subdimensions were scored as mean values. Self-concordance was calculated as summed mean score difference.
Future perspective on running intention & behavior			
Intention strength	Subjective evaluation of how strong the intention is to run regularly in the coming weeks and months.	(71)	Single item scales (10-point Likert scale)
Effort readiness	Subjective evaluation of how much effort one is willing to muster up to run regularly in the coming weeks and months.	(71)	Single item scales (10-point Likert scale)

IEC, implicit-explicit concordance; IED, implicit-explicit discrepancy.

### 2.3 Materials

#### 2.3.1 Implicit associations

**Figure 1:**
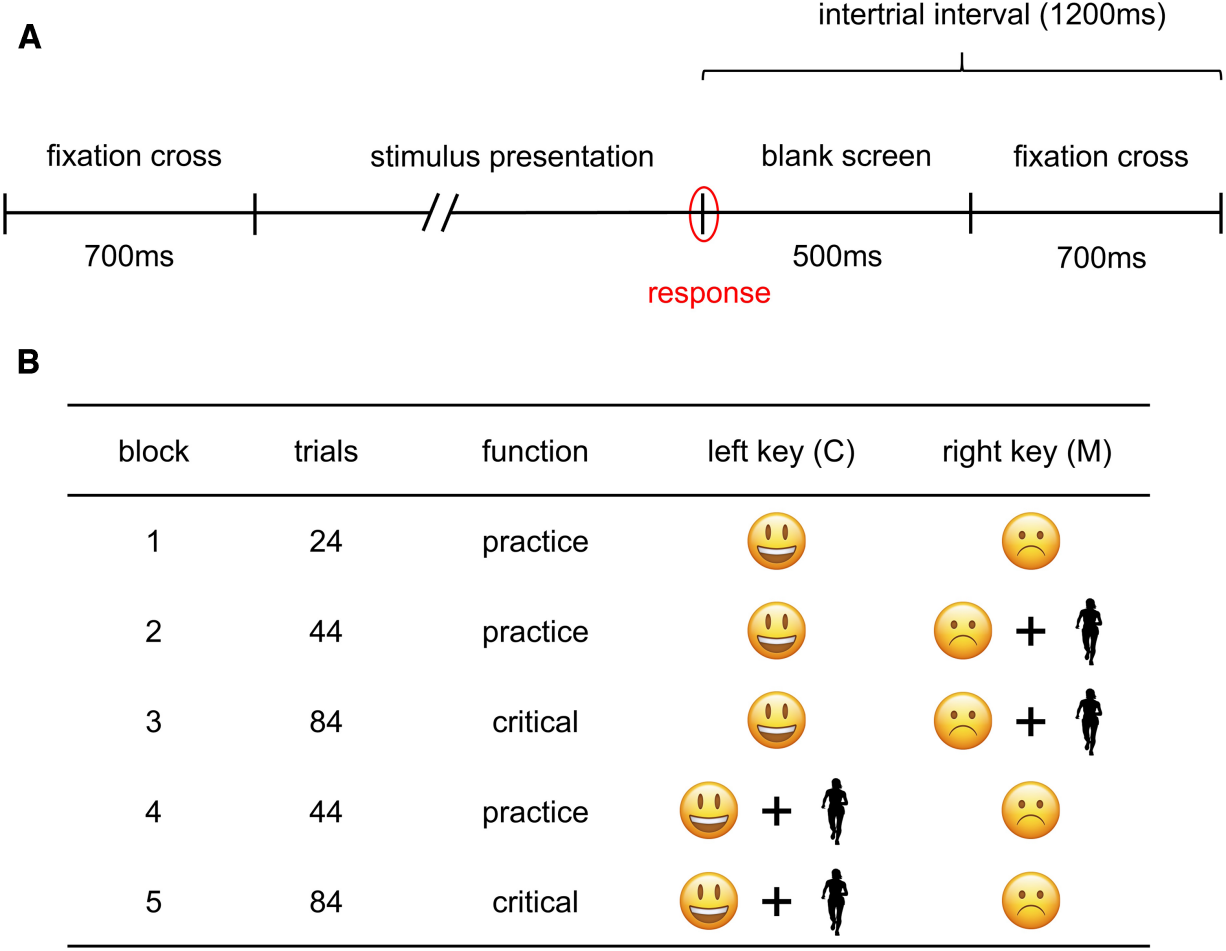
Illustration of trial structure (**A**) and task procedure (**B**) of the ST-IAT.

Implicit affective associations of female runners were measured via a Single-Target Implicit Association Test (ST-IAT) (51). We chose the ST-IAT for following reasons: (1) Being a derivate of the Implicit Association Test (IAT) (52), the ST-IAT assumes that past experiences with a target concept (e.g., running) influence the affective evaluation (positive vs. negative) of that concept on an implicit level (i.e., implicit affective association towards running) (53). Theoretical models in the area of exercise and physical activity behavior assume that experienced affect during exercise or physical activity is decisive for the formation of implicit associations and subsequent behavior like regular running behavior (29, 31). (2) In contrast to the original IAT, the ST-IAT attempts to measure absolute implicit associations towards a single concept (i.e., running). (3) The ST-IAT mainly demonstrates satisfying internal consistency (IC) in the area of exercise and physical activity (54–56), which is comparable to other research areas and outperforms most alternative implicit measures (57, 58). 4) The ST-IAT has been successfully used to predict self-reported (55) and objectively measured physical activity (56, 59, 60) independent of explicit processes. Four distinct dark silhouette images of female runners were utilized as the target category “running” to minimize potential confounding visual aspects related to body image, appearance, clothing, or facial expression (61). A frontal perspective of the runners was chosen to avoid response bias by directional encoding lateralization (62, 63). To represent positive and negative affective categories, we selected four smileys and frownies each from the iOS operating system based on subjective ratings from the Lisbon Emoji and Emoticon Database (LEED) (64). Stimulus sizes were standardized on a 15.6-inch laptop monitor (HP EliteBook 850 G5, HP Inc., Palo Alto, CA, USA) with a resolution of 1,920 × 1,080 (see Supplementary Material 1). Participants were instructed to associate stimuli with their assigned categories (smileys = positive, frownies = negative, female runners = running) by pressing designated keys. Participants pressed the M key for stimuli associated with categories on the right side and the C key for categories on the left side. Affective category labels (negative on M, positive on C) were kept constant throughout the task. Target category assignment followed the association condition of the block, aligning with positive or negative affective categories (see Figure 1B). Labels served as reminders during trials, positioned on the upper right and left screen side relative to the stimulus. The ST-IAT comprised five blocks (B1 to B5): three practice blocks (B1, B2, and B4) and two critical blocks (B3 and B5). In B1, participants were familiarized with the task featuring only affective categories (50:50; 24 trials). The subsequent four blocks (B2 to B5) featured stimuli of the target category (female runners) and associated or unassociated affective categories based on the block-specific association condition. Blocks B2 and B3 represented the negative association condition, and B4 and B5 the positive association condition. Critical blocks (B3 and B5) included 84 trials and were preceded by practice blocks (B2 and B4) with 44 trials, following Nosek et al. (61) to mitigate block order effects. Stimuli were presented in a randomized order, and to prevent response bias, an adjusted left-right hand ratio was implemented in B2 to B5, with a ratio of 12:12:20 in practice blocks and 24:24:36 in critical blocks, following Karpinski and Steinmann's approach (65). Each trial started with a central fixation cross for 700 ms, immediately followed by stimulus onset. Stimuli remained on screen in central position until a response was given (i.e., key press onset). Responses were followed by a feedback screen (i.e., green check symbol for correct and red cross symbol for incorrect responses) in B1 and a blank screen B2–B5 for 500 ms. Intertrial interval amounted to 1,200 ms (see Figure 1A). Response times (RT) were calculated from stimulus onset to key press onset. A score indicating implicit affective associations towards running was calculated as follows: (1) Participants with trials <300 ms in more than 10% of included trials were excluded from further analysis (66). (2) Trials from both practice and critical blocks (B2 to B5) were included for scoring (66). No distinction was being made between trials of the practice and critical block of the respective association conditions (67). (3) Extreme RTs were treated by 10% statistical winsorizing. This approach accounts for the influence of extremely fast or slow RT without discarding trials while considering individual RT tendencies. Further, applying 10% statistical winsorizing outperformed other extreme RT treatment methods for IAT data with no-built error penalty in terms of reliability and validity (67). (4) Untreated error trials were included in scoring as they might include relevant information regarding a person's implicit association (67). (5) The ST-IAT score was then calculated as mean gaussian rank RT difference (i.e., the G-Score). The G-Score is a scale invariant, non-parametric scoring algorithm, which was originally developed as an alternative to the D-Score for the Brief Implicit Association Test (BIAT) (68) and shows convincing reliability and validity for the IAT (67). Sriram and colleagues (69) argued that the G-Score might be superior to the often used D-Score since its more robust against extreme RT influence and non-linear transformations of RT data (i.e., treatment of extreme RT and errors). To calculate the G-Score, first, all trials of a task performance included for scoring were combined in one data set regardless of association condition and received a fractional rank (percentile) from the fastest to the slowest RT. Then, the percentiles were standardized and means of the standardized percentiles per association condition were calculated. The G-Score was computed as the difference of these means so that a positive score indicated a positive implicit association towards running and a negative score indicated a negative implicit association towards running (68). The IC of the used ST-IAT was calculated as stratified Monte Carlo split reliability (*r* = .88) to account for confounding effect of trial splitting (i.e., confounds with time, task design, trial sampling and non-linear scoring). Stratification was based on association condition (i.e., positive and negative association) and stimulus type (i.e., smiley, frowny and runner). Resampling of trials with replacement was run with 10,000 replications. Then a simple mean of the correlation coefficients was taken (70).

#### 2.3.2 Explicit associations

An 11-point Feeling Thermometer (FT) (“How do you feel while running? Please rate your feeling on the scale below.”) was used to measure explicit affective associations towards running. The measure ranged from 0 (unpleasant/negative) to 10 (pleasant/positive), whereas 5 indicated a neutral feeling (66).

#### 2.3.3 Past perspective on running intention and behavior

Participants were asked whether they were engaged in regular running in the past and intend to (re)start running on a regular basis. Participants were instructed to choose one out of five possible responses: (1) “I have never run on a regular basis and do not intend to start.” (2) “I used to run on a regular basis, but do not currently intend to start again.” (3) “I have never run on a regular basis, but I am considering starting.” (4) “I used to run on a regular a basis and I am considering getting back into it.” (5) “I run on a regular basis.”

#### 2.3.4 Present perspective on running intention and behavior

Current running behavior was assessed via self-report. Participants were classified as non-runners, irregular runners (<1 run/week) and regular runners (≥1 run/week) based on the last 6 months.

Current motivational aspects towards regular running were assessed with a modified version of the German-language sport- and exercise-related self-concordance scale (SSK-scale) from Seelig and Fuchs (71). All 12 original items were used and reformulated in order to assess running-related self-concordance. The items were preceded by an information text emphasizing that the items refer to regular running. Items ranged on a 6-point scale from 1 “is not true at all” to 6 “is exactly true”. The SSK subscales measuring intrinsic (α = .92), identified (α = .90), introjected (α = .77) and extrinsic (α = .79) motivation towards running as well as the SSK-index were used for analysis. SSK subscales were calculated by taking the mean of corresponding items. The SSK-index representing self-concordance was calculated by subtracting the sum of the introjected and extrinsic SSK subscales from the sum of the intrinsic and identified subscales.

#### 2.3.5 Future perspective on running intention and behavior

Participants’ intention strength and effort readiness to engage in running on a regular basis in the future were assessed with two modified 10-point scales from Seelig & Fuchs (71). The running-related intention strength item reads: “How strong is your intention to engage in regular running in the next weeks and months?” (1 = “not strong at all” to 10 = “very strong”). The running-related effort readiness item reads: “How much effort would you be willing to muster up in order to engage in regular running in the next weeks and months?” (1 = “none at all” to 10 = “huge amount”).

#### 2.3.6 Main exercise

To quantify how much participants were focused on running, they were asked to indicate which exercise they mainly do. In addition, this information allowed conclusions about whether participants were generally physically active.

### 2.4 Statistical analyses

**Figure 2:**
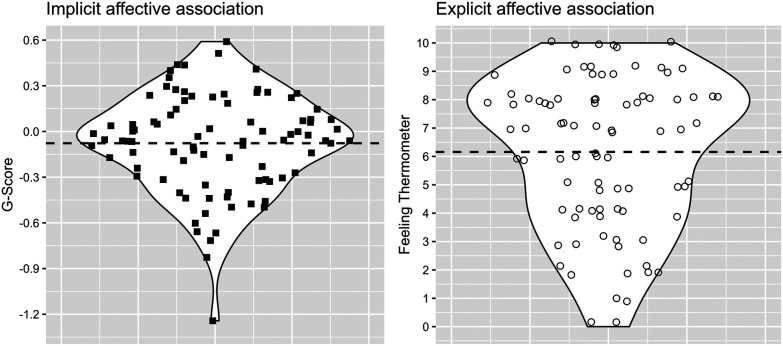
Illustration of distributional information of implicit and explicit affective association variables as violin plots (mean values are displayed as dashed horizontal lines).

**Figure 3:**
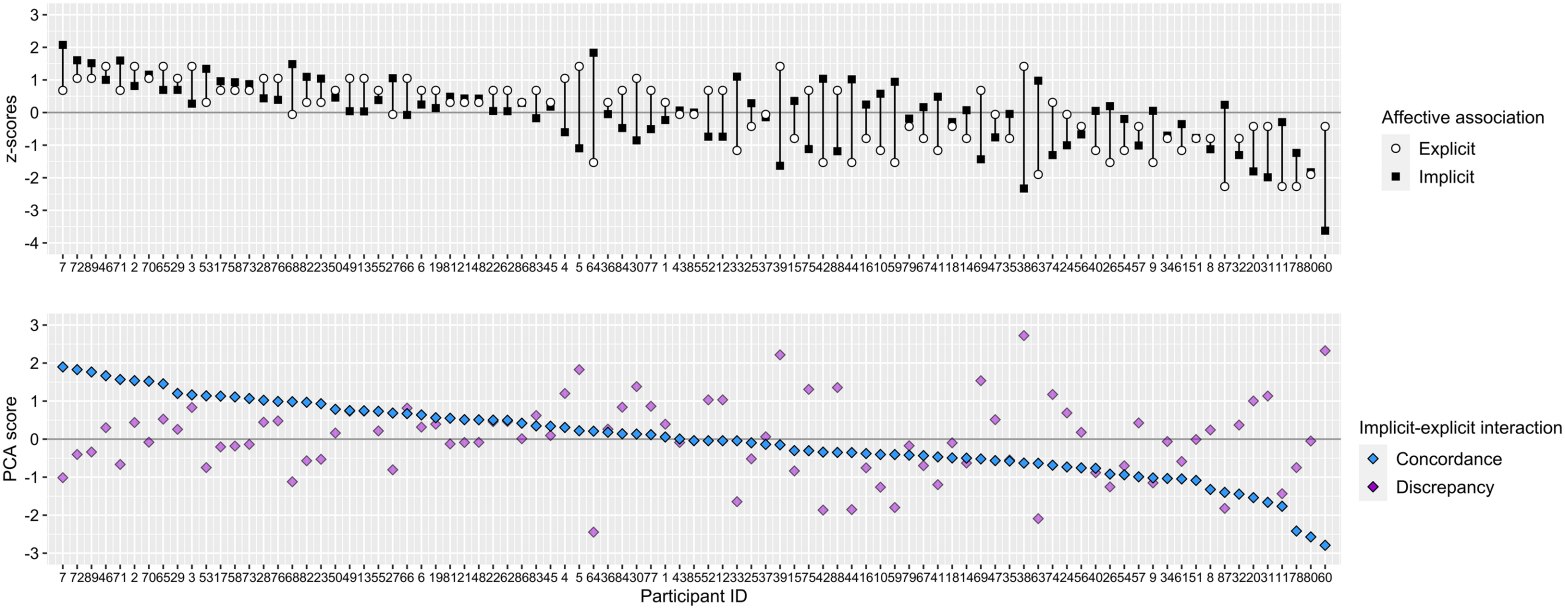
Illustration of implicit-explicit interaction sorted by IEC scores associations in descending order and the corresponding z-standardized implicit and explicit (associations *n* = 89). PCA, principal component analysis.

**Figure 4:**
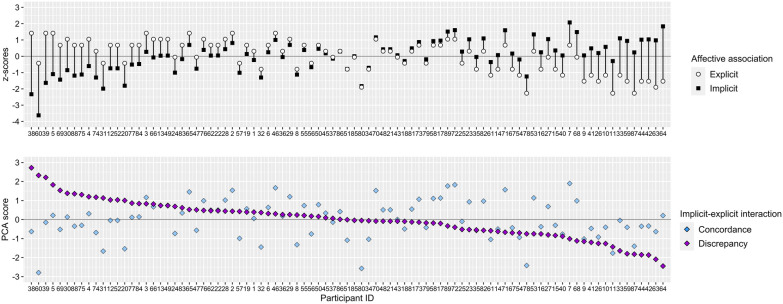
Illustration of implicit-explicit interaction sorted by IED scores in descending order and the corresponding z-standardized implicit and explicit associations (*n* = 89). PCA, Principal Component Analysis.

**Figure 5:**
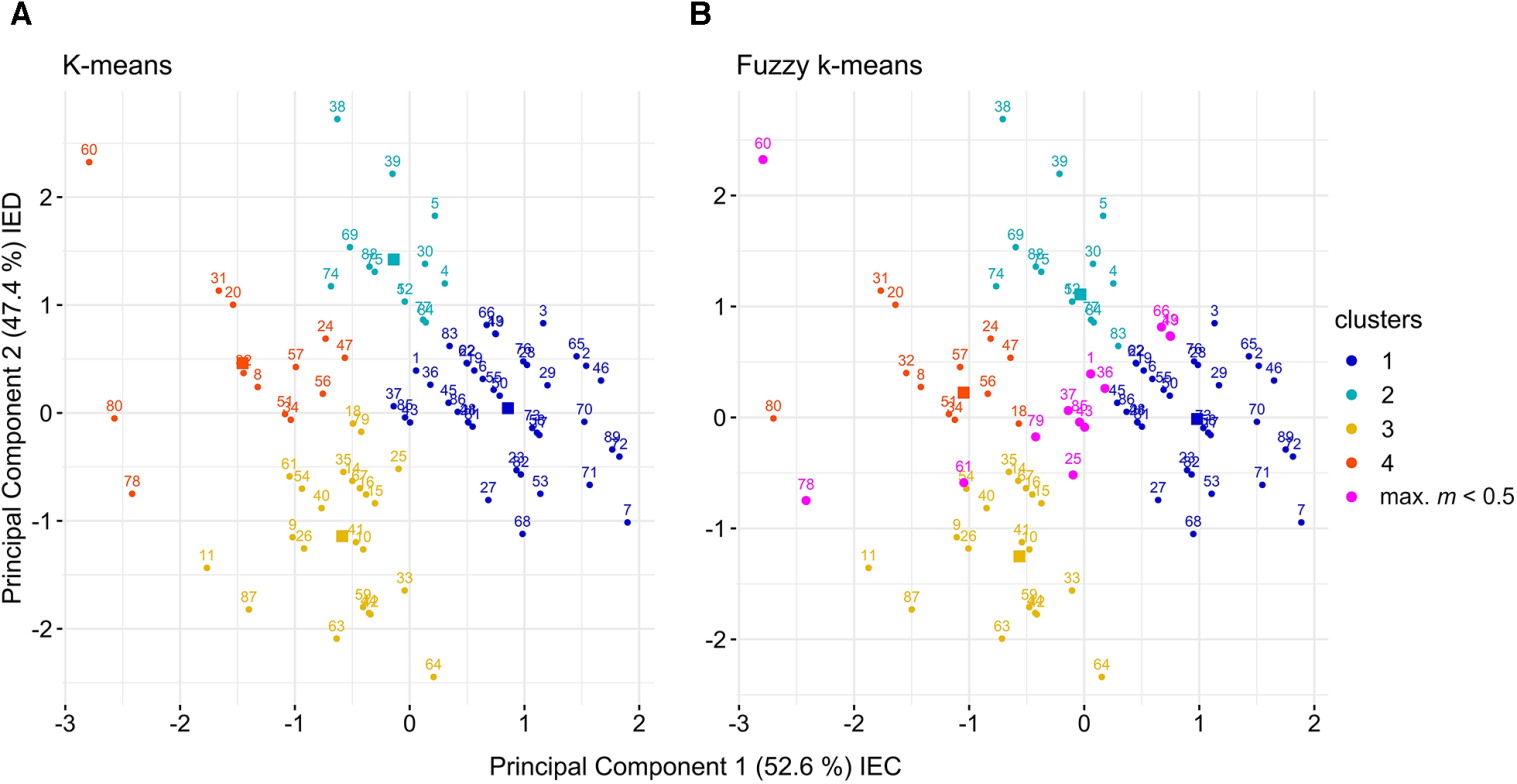
Illustration of k-means (**A**) and fuzzy k-means (*m* = 2.0) (**B**) cluster solutions (k = 4) on their principal components indicating participant IDs, cluster centers and observations with unclear cluster membership (max. *m* < 0.5). IEC, implicit-explicit concordance; IED, implicit-explicit discrepancy. Cluster centers are depicted as rectangles.

**Figure 6:**
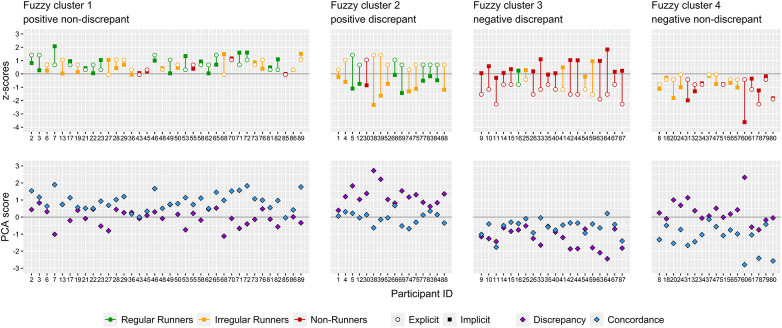
Illustration of implicit-explicit interaction and current running behavior grouped by fuzzy k-means cluster solution (k = 4, *m* = 2.0).

In accordance with Brand and Antoniewicz (39) principal component analysis (PCA) was used on implicit (G-Score) and explicit association (Feeling Thermometer) variables (see Figure 2) to extract two component scores representing the combined valence (i.e., IEC) and the difference (i.e., IED) of implicit and explicit affective associations towards running. Implicit and explicit raw values are z-standardized as part of the PCA scoring procedure. This allows an adequate representation of the interactive implicit-explicit relationship taking different scale ranges into account. Note that standardized IEC and IED scores must be interpreted relative to the sample. Positive IEC values indicate that both implicit and explicit affective associations are rather positive relative to the sample and vice versa. Positive IED values indicate an implicit-explicit discrepancy with values of explicit associations being more positive than implicit associations relative to the sample and vice versa. Both Figures 3, 4 show how implicit and explicit affective associations are related to their IEC and IED values for each participant. In Figure 3 the relation of IEC values to their corresponding implicit and explicit association values is emphasized by arranging IEC values from left to right in descending order. In Figure 4 the same data is illustrated with an emphasis on IED. Variables of implicit and explicit affective associations, their interaction scores (i.e., IEC and IED) as well as variables of running behavior operationalized by a past, present and future perspective were illustrated by descriptive statistics (see Table 3). Then, k-means clustering (72) was performed on IEC and IED values based on squared Euclidean distances with 25 random starts and a maximum of 1,000 iterations allowed per set. Prior to applying the k-means a dendrogram was derived from Ward's hierarchical clustering to determine the number of clusters. The dendrogram indicated solutions of three or four clusters suitable. Inspection of the resulting patterns of IEI from the k-means clusters implied that four clusters were best in terms of interpretability (Supplementary Material 3). Further visual inspection of the four k-means clusters plotted in relation to their principal components indicated potential outliers and overlap between clusters (Figure 5). Thus, fuzzy k-means clustering with k = 4 was run on IEC and IED (73). Number of random starts and maximum iterations were kept consistent to the crisp k-means solution. As commonly used, the membership exponent *m* was set to 2.0 (74). The four fuzzy clusters were described by their observed IEI patterns, cluster membership overlap (see Tables 4 and 5) and compared by variables related to past, present and future running behavior. Clusters were labeled according to their prevalent IEI patterns (see Table 6 and Figure 6). Due to *n* < 5 in some clusters Fisher's exact tests were utilized to detect difference in past running experience and current running behavior (75). Shapiro–Wilk tests detected several significant violations of normality for motivational and intentional variables in some clusters. Homogeneity of variance between clusters was not given for identified motivation. Further distributional information can be found in Supplementary Material 3. To ensure consistency and statistical rigor, we computed non-parametric univariate Kruskal–Wallis tests to analyze cluster differences for each motivational and intentional variable, given the small cluster sample sizes. Effect sizes of *η*^2^ were interpreted according to Cohen (76). Statistical significance was assumed at *p* = 0.05 and *post-hoc* Bonferroni corrected Wilcoxon tests were calculated for significant differences. All statistical analyses were conducted using the RStudio software. ST-IAT scores and internal consistency were computed with a customized R script. PCA-based IEC and IED scores were calculated using the *psych* package (77). Fisher's exact tests were calculated using the *CrossTable* function from the *gmodels* package (78). Inferential statistical tests (e.g., Kruskal–Wallis tests) were conducted using the *rstatix* package (79). K-means cluster analysis was computed using the *kmeans* function from the *stats* package (80). The *FKM* function from the *fclust* package was used to perform fuzzy k-means clustering (81).

**Table 3:** Descriptive statistics of implicit and explicit affective associations, motivational and intentional variables (*n* = 89).

	*M*	*SD*	Range	Skew	Kurt.
Affective Associations					
Implicit	−0.08	0.32	−1.24–0.59	−0.65	3.93
Explicit	6.16	2.71	0.00–10.00	−0.53	2.29
IEC	0.00	1.00	−2.79–1.9	−0.36	3.02
IED	0.00	1.00	−2.45–2.72	0.03	3.15
Motivation					
Intrinsic motivation	3.31	1.62	1.00–6.00	0.02	1.63
Identified motivation	4.41	1.41	1.00–6.00	−0.80	2.69
Introjected motivation	3.13	1.22	1.00–5.67	0.13	2.44
Extrinsic motivation	1.43	0.79	1.00–4.33	2.11	6.66
Self-concordance (SSK-index)	3.15	2.34	−2.00–8.00	−0.10	2.15
Intention					
Intention strength	5.84	3.23	1.00–10.00	−0.20	1.56
Effort readiness	6.19	2.58	1.00–10.00	−0.42	2.28

IEC, implicit-explicit concordance; IED, implicit-explicit discrepancy; Kurt., kurtosis.

**Table 4:** Percentage of fuzzy k-means solutions that belong to corresponding k-means clustering solutions (*m* = 2.0).

	K-means cluster 1	K-means cluster 2	K-means cluster 3	K-means cluster 4
Fuzzy cluster 1 [%]	**100**	0.00	0.00	0.00
Fuzzy cluster 2 [%]	18.8	**81**.**2**	0.00	0.00
Fuzzy cluster 3 [%]	0.00	0.00	**100**	0.00
Fuzzy cluster 4 [%]	5.9	0.00	17.6	**76**.**5**

The percentage of fuzzy cluster observations that are comprised in their direct k-means cluster counterparts is in bold.

**Table 5:** Mean cluster membership in percentage for fuzzy k-means (*m* = 2.0).

	% cluster 1	% cluster 2	% cluster 3	% cluster 4
Cluster 1	**69** **.** **4**	14.9	7.9	7.8
Cluster 2	10.5	**75**.**8**	3.6	10.2
Cluster 3	7.7	5.3	**73**.**6**	13.5
Cluster 4	7.5	14.9	12.9	**64**.**8**

The mean membership degree of the observations in each fuzzy cluster is in bold.

**Table 6:** Summary of affective associations and variables related to running and exercise behavior for fuzzy k-means clusters (k = 4). Variables are described by *M (SD)* unless indicated differently.

	Cluster 1	Cluster 2	Cluster 3	Cluster 4
	Positive non-discrepant	Positive discrepant	Negative discrepant	Negative non-discrepant
Cluster size *n*^a^	36 (5)	16 (2)	20 (1)	17 (5)
Affective associations				
Implicit				
Raw score	0.14 (0.18)	−0.37 (0.19)	0.07 (0.17)	−0.43 (0.28)
z-score	0.67 (0.56)	−0.91 (0.60)	0.45 (0.54)	−1.11 (0.86)
Explicit				
Raw score	7.97 (1.18)	8.44 (0.96)	2.65 (1.35)	4.29 (1.65)
z-score	0.67 (0.44)	0.84 (0.36)	−1.29 (0.50)	−0.69 (0.61)
IEC	0.93 (0.50)	−0.05 (0.38)	−0.58 (0.46)	−1.24 (0.77)
IED	0.00 (0.50)	1.27 (0.59)	−1.27 (0.57)	0.31 (0.72)
Intrinsic motivation	4.20 (1.25)	4.52 (1.18)	1.68 (0.88)	2.18 (0.92)
Identified motivation	5.14 (0.78)	5.48 (0.53)	2.93 (1.45)	3.59 (1.04)
Introjected motivation	3.41 (0.89)	4.04 (1.18)	2.35 (1.32)	2.59 (0.98)
Extrinsic motivation	1.52 (0.81)	1.19 (0.52)	1.57 (1.01)	1.33 (0.68)
Self-concordance	4.42 (1.69)	4.77 (1.59)	0.70 (1.12)	1.84 (2.12)
Intention strength	7.56 (2.51)	8.00 (2.16)	3.10 (2.73)	3.41 (1.87)
Effort readiness	7.42 (1.84)	7.81 (1.68)	4.25 (2.67)	4.35 (1.93)

IEC, implicit-explicit concordance; IED, implicit-explicit discrepancy.

^a^ Number of observations with max. *m* < 0.5 in parentheses.

## 3 Results

### 3.1 Description of implicit and explicit affective associations

Descriptive statistics indicate that a broad range of implicit (range = −1.24–0.59) and explicit (range = 0.00–10.00) affective associations towards running were manifested by the sample. On average participants showed rather neutral affective associations towards running on the implicit (*M* = −0.08, *SD* = 0.32) and explicit (*M* = 6.16, *SD* = 2.71) level with a slight tendency towards positive values (skew_implicit_ = −0.65, skew_explicit_ = −0.53) (see Table 3 and Figure 2). IEC scores >0 indicate that a participant's implicit and explicit associations are both rather positive and IEC scores <0 indicate that a participant's implicit and explicit associations are both rather negative. This relationship is impacted by the extent, yet not by the direction, of the difference between implicit and explicit associations (see Figure 3). Figure 4 shows that IED scores close to zero correspond to small differences between implicit and explicit associations. IED scores >0 correspond to implicit-explicit differences characterized by explicit associations being more positive than implicit associations and IED scores <0 relate to explicit associations being more negative than implicit associations. IED scores are sensitive to the direction and the extent of difference between implicit and explicit associations. This relationship is irrespective of the valence of implicit and explicit associations (i.e., IEC).

### 3.2 Description of running behavior

34 (38.3%) participants stated to have past experience with regular running. Out of this sample 23 (67.6%) participants considered to re-engage in regular running, while 11 (32.4%) did not. 28 (31.5%) participants did not have any prior experience with regular running. Out of these, 13 (46.4%) expressed their intention to start regular running in the future, while 15 (53.6%) did not. 27 (30.3%) participants indicated to be engaged in regular running behavior. In terms of current running behavior, 25 (28.1%) participants reported that they had run at least once a week for the past six months (i.e., regular runners). 34 (38.3%) participants reported doing less than one running session per week (i.e., irregular runners) and 30 (33.7%) participants stated to have not been running at all during the last six months (i.e., non-runners). Intrinsic motivation was moderate overall (*M* = 3.31, *SD* = 1.62). Identified motivation was quite high (*M* = 4.41; *SD* = 1.41). Introjected motivation was moderate (*M* = 3.13, *SD* = 1.22). Extrinsic motivation was rather low (*M* = 1.43, *SD* = 0.79). Self-concordance ranged from −2.00–8.00 and was slightly positive (*M* = 3.15, *SD* = 2.34). Both intention strength (*M* = 15.84, *SD* = 3.23) and effort readiness (*M* = 6.19, *SD* = 2.58) showed moderate values overall. Distributions of motivational and intentional variables demonstrate low values of skew and kurtosis, except for extrinsic motivation which is left skewed and shows large kurtosis (see Table 3).

### 3.3 Description of main exercise

Three (3.4%) participants made no indications about any main exercise that they currently pursuit, and, thus, were considered as physically inactive. Six (6.7%) participants stated that running is currently their main exercise, while 80 (89.9%) participants considered exercises other than running their main exercise.

### 3.4 Fuzzy clusters

Fuzzy k-means clustering resulted in a four-cluster structure similar to the crisp k-means solution (Rand index = 0.76). Fuzzy clusters were numbered so that they would correspond to their crisp k-means counterparts (see Figure 5). Table 4 shows similarities between the four clusters solutions of k-means and fuzzy k-means. Fuzzy clusters share most observations with their crisp k-means counterparts (see Table 3 diagonal). Fuzzy clusters 1 and 3 are 100% comprised in k-means clusters 1 and 3, while 18.8% and 23.5% of observations of fuzzy clusters 2 and 4, were assigned to different k-means clusters than their corresponding k-means clusters. Thus, k-means and fuzzy k-means produced similar, yet slightly different cluster solutions. Comparison of the four clusters were based on the fuzzy cluster solution.

### 3.5 Cluster description

Fuzzy cluster 1 was named “positive non-discrepant” cluster (*n* = 36) and showed positive IEC (*M* = 0.93, *SD* = 0.50) and small IED (*M* = 0.00, *SD* = 0.50). Hence, implicit and explicit affective association are rather congruent (i.e., small discrepancies) and high in valence (i.e., positive IEC). Five participants have a maximum membership degree < 0.5 (mean membership degree = 69.4%). Table 5 shows a 14.9% membership overlap with fuzzy cluster 2 and similar overlaps with fuzzy clusters 3 and 4 (7.9% and 7.8%). 47.2% of the participants in the “positive non-discrepant” cluster 1 are regular runners (*n* = 17), 38.9% are irregular runners (*n* = 14) and 13.9% are non-runners (*n* = 5). 15 non-regular runners reported to have experience with regular running, while two did not. High scores of intrinsic (*M* = 4.20, *SD* = 1.25) and identified motivation (*M* = 5.14, *SD* = 0.78) on average were found. Introjected motivation was moderate (*M* = 3.41, *SD* = 0.89) and extrinsic motivation was low (*M* = 1.52, *SD* = 0.81). Self-concordance was positive (*M* = 4.42, *SD* = 1.69). Both intention strength (*M* = 7.56, *SD* = 2.51) and effort readiness (*M* = 7.42, *SD* = 1.84) were rather high.

Fuzzy cluster 2 was named “positive discrepant” cluster (*n* = 16) and demonstrated IEC values close to zero (*M* = −0.05, *SD* = 0.38) and positive IED (*M* = 1.27, *SD* = 0.59). Implicit and explicit affective associations are rather distant in valence from each other with explicit associations being more positive than implicit associations. Two observations have a maximum membership degree <0.5 (mean membership degree = 75.8%). “Positive discrepant” cluster 2 overlapped with both clusters 1 and 4 to a similar amount (10.5% and 10.2%) and to a small degree with cluster 3 (3.6%). Regular runners made up 43.8% (*n* = 7) and irregular runners 50.0% (*n* = 8) of observations in the “positive discrepant” cluster 2. Non-runners were represented by one observation (6.2%). Of non-regular runners 7 reported to have experience with regular running and three did not. Intrinsic (*M* = 4.52, *SD* = 1.18), identified (*M* = 5.48, *SD* = 0.53) and introjected motivation (*M* = 4.04, *SD* = 1.18) were moderate to high on average and extrinsic motivation was low (*M* = 1.19, *SD* = 0.52). Self-concordance was moderately positive (*M* = 4.77, *SD* = 1.59). Intention strength (*M* = 8.00, *SD* = 2.16), effort readiness (*M* = 7.81, *SD* = 1.68) were remarkably high.

Fuzzy cluster 3 was named “negative discrepant” cluster (*n* = 20). Both IEC (*M* = −0.58, *SD* = 0.46) and IED (*M* = −1.27, *SD* = 0.57) were mainly negative. Implicit and explicit affective associations are rather distant in valence from each other with explicit associations being more negative than implicit associations. One observation has a maximum membership degree <0.5 (mean membership degree = 73.6%). Cluster overlaps amounted to 7.7%, 5.3% and 13.5% with clusters 1, 2 and 4. One regular runner (5.0%), 4 irregular runners (20.0%) and 15 non-runners (75.0%) were assigned to the “negative discrepant” cluster 3. 7 non-regular runners reported to have past experience with regular running in the past, while 11 did not. Intrinsic (*M* = 1.68, *SD* = 0.88) and extrinsic motivation (*M* = 1.57, *SD* = 1.01) were low. Identified (*M* = 2.93, *SD* = 1.45) and introjected motivation (*M* = 2.35, *SD* = 1.32) were moderate. Self-concordance was neutral (*M* = 0.70, *SD* = 1.12). Intention strength (*M* = 3.10, *SD* = 2.73) and effort readiness (*M* = 4.25, *SD* = 2.67) showed moderate values on average.

Fuzzy cluster 4 was named “negative non-discrepant” cluster (*n* = 17). IEC values were negative (*M* = −1.24, *SD* = 0.77) and IED slightly positive (*M* = 0.31, *SD* = 0.72). Implicit and explicit affective associations are rather congruent (i.e., small discrepancies) and low in valence (i.e., negative IEC). Five observations have a maximum membership degree <0.5 (mean membership degree = 64.8%). Cluster overlaps amounted to 7.5%, 14.9% and 12.9% with clusters 1, 2 and 3. No regular runner was assigned to the “negative non-discrepant” cluster 4, while irregular runners (*n* = 8) and non-runners (*n* = 9) made up 47.1% and 52.9%. 5 non-regular runners reported to have run regularly in the past, while 12 did not. Intrinsic (*M* = 2.18, *SD* = 0.92) and extrinsic motivation (*M* = 1.33, *SD* = 0.68) were low. Identified (*M* = 3.59, *SD* = 1.04) and introjected motivation (*M* = 2.59, *SD* = 0.98) were moderate. Self-concordance was neutral (*M* = 1.84, *SD* = 2.12). Intention strength (*M* = 3.41, *SD* = 1.87) and effort readiness (*M* = 4.35, *SD* = 1.93) showed overall moderate values.

### 3.6 Cluster comparison

**Figure 7:**
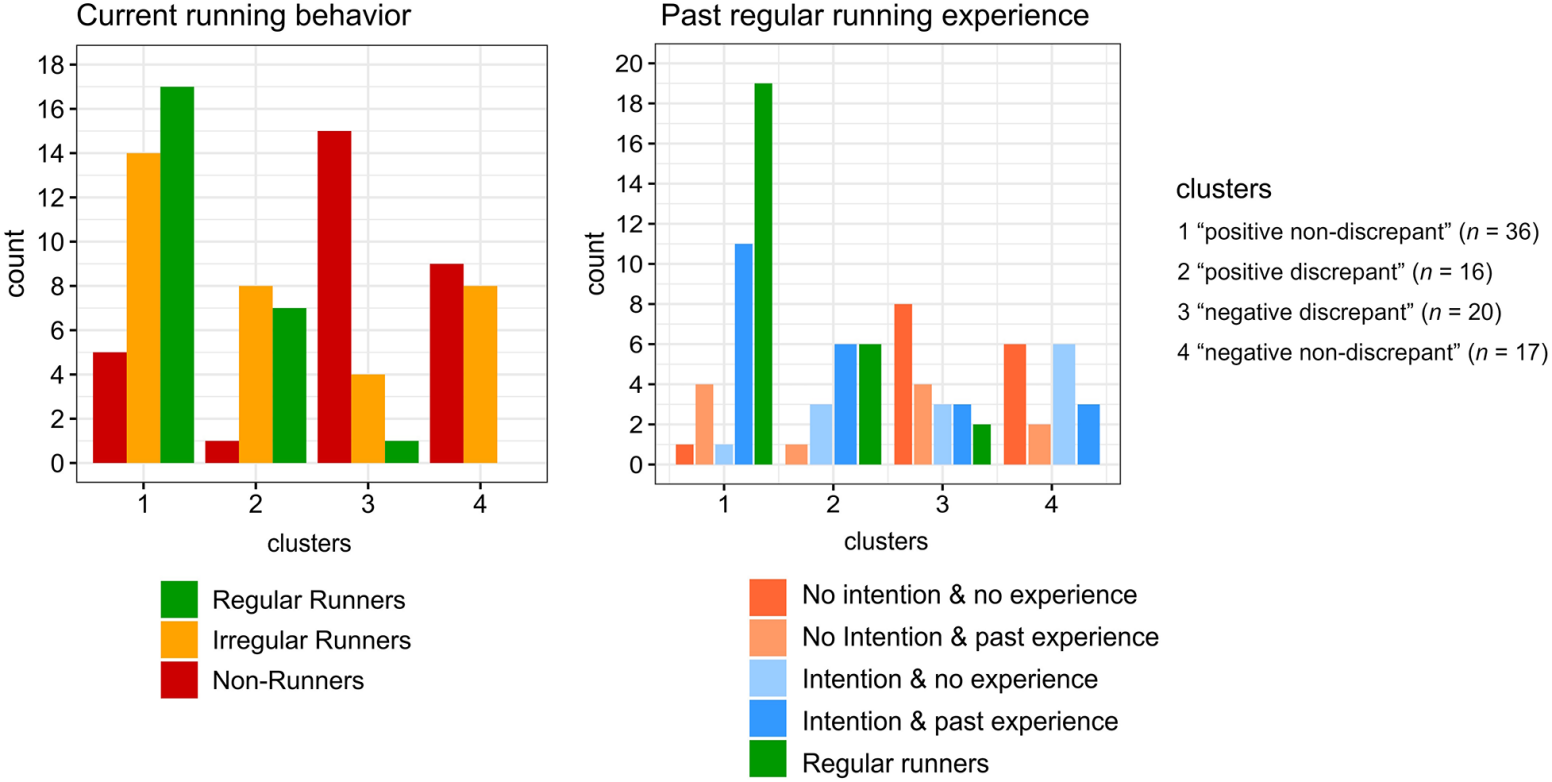
Bar charts displaying frequency distributions of current running behavior and past regular running experience.

**Figure 8:**
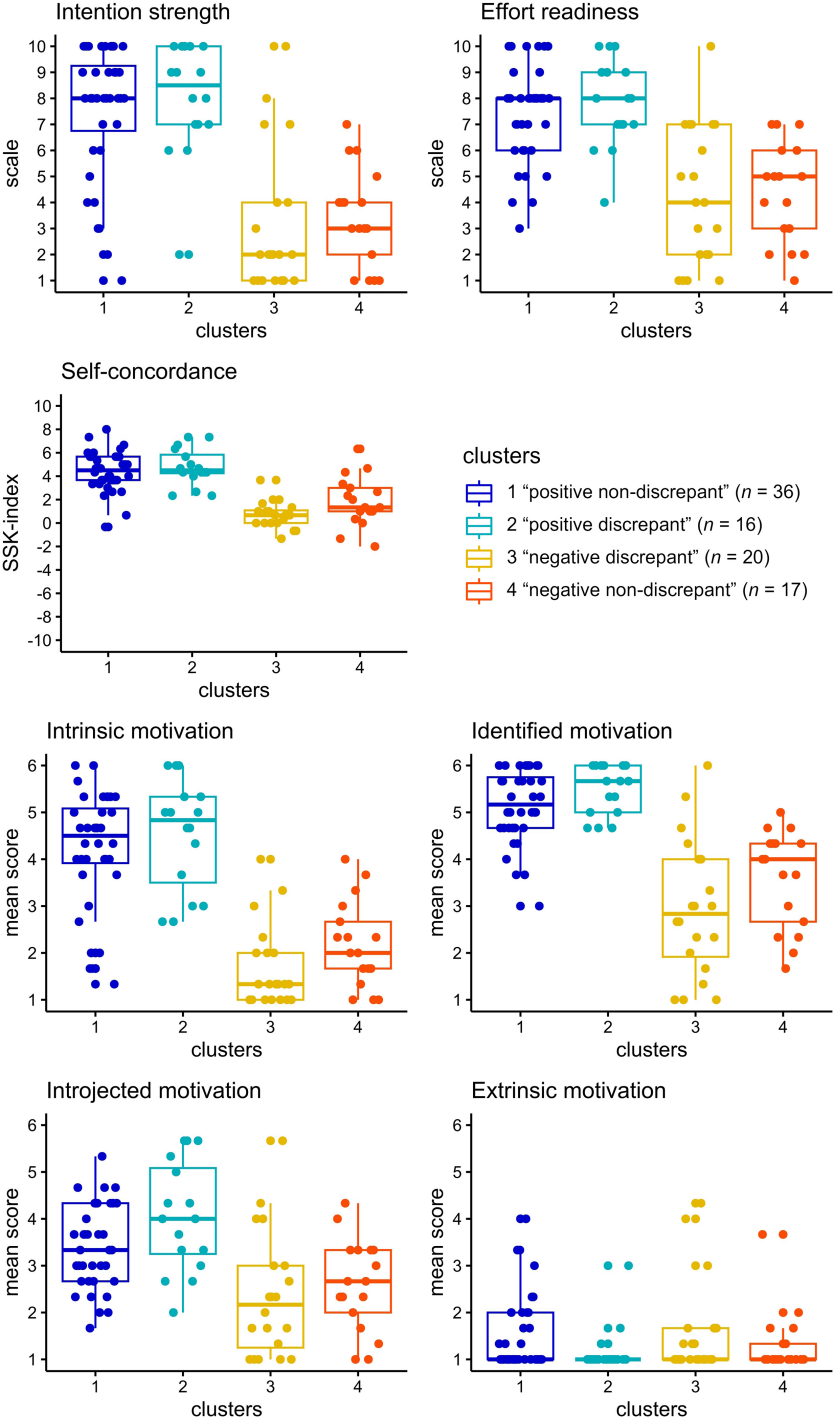
Boxplots of motivational and intentional variables grouped by fuzzy clusters.

Fisher's exact tests revealed significant differences in frequency distributions between clusters for past regular running experience [*χ*^2^(9) = 22.6, *p* = .004] and current running behavior [*χ*^2^(6) = 37.1, *p* < .001] (see Figure 7). Both “positive non-discrepant” cluster 1 and “positive discrepant” cluster 2 contain largely regular and irregular runners and few non-runners. In contrast, “negative discrepant” cluster 3 constitutes mainly non-runners and only few regular and irregular runners. “Negative non-discrepant” cluster 4 is exclusively composed of almost equal amounts of irregular runners and non-runners. Regarding past regular running experience of non-regular runners, “positive non-discrepant” cluster 1 and “positive discrepant” cluster 2 feature mainly participants who report to have past experience with regular running and most are currently considering starting to run regularly. In “negative discrepant” cluster 3 the majority of participants report to have no experience with regular running and currently do not consider starting. Participants with past regular running experience are divided in those who are considering starting to run regularly and those who are not. Most participants assigned to “negative non-discrepant” cluster 4 reported to have no experience with regular running. Participants who are considering starting to run regularly and those who are not were almost equally represented among those with and without past regular running experience. Kruskal-Wallis tests indicated significant differences in intrinsic motivation [*H*(3) = 48.2, *p* < .001, *η*^2^ = 0.53], identified motivation [*H*(3) = 45.9, *p* < .001, *η*^2^ = 0.51], introjected motivation [*H*(3) = 21.5, *p* < .001, *η*^2^ = 0.22], running-related self-concordance [*H*(3) = 45.5, *p* < .001, *η*^2^ = 0.50], intention strength [*H*(3) = 39.5, *p* < .001, *η*^2^ = 0.43] and effort readiness [*H*(3) = 34.8, *p* < .001, *η*^2^ = 0.37] between clusters. No significant differences between clusters were found for extrinsic motivation [*H*(3) = 3.4, *p* = .331, *η*^2^ = 0.01]. Pairwise Bonferroni adjusted Wilcoxon tests revealed that both the “positive non-discrepant” cluster 1 and “positive discrepant” cluster 2 exhibited significantly higher scores for intrinsic and identified motivation, self-concordance, intention strength and effort readiness than the “negative discrepant” cluster 3 and “negative non-discrepant” cluster 4. No significant differences for these variables were found between clusters 1 and 2 as well as 3 and 4. For introjected motivation both the “positive non-discrepant” cluster 1 and “positive discrepant” cluster 2 displayed each significantly higher values than the “negative discrepant” cluster 3. Further, “positive discrepant” cluster 2 had significantly higher scores in introjected motivation than “negative non-discrepant” cluster 4. In Figure 8 cluster differences are illustrated by boxplots for motivational and intentional variables.

## 4 Discussion

Previous studies have attempted to distinguish individuals with different running behaviors by their implicit and explicit associations (49). In the present study patterns of implicit and explicit affective association towards running were identified in young women. Four clusters containing meaningful configurations of IEC and IED were found. To our knowledge, fuzzy k-means clustering has been used for the first time in exercise psychology. Values of mean cluster membership degree indicate unique IEI core patterns for each identified cluster. Differences in running behavior as well as in motivational and intentional variables between clusters emphasize the relevance of distinct IEI patterns in understanding affective processes in young female runners and non-runners. Looking at the distribution of regular runners and non-runners across all four clusters, it becomes apparent that positive affect is important in successfully implementing regular running behavior (see Figure 6 and Table 6). However, due to the cross-sectional design of the study it remains unclear whether overall positive affect (i.e., IEC) contributes to the process to run regularly or is simply an indicator of regular running behavior. Furthermore, differences in introjected motivation, intention strength and effort readiness suggest that effortful explicit self-regulatory processes are needed to implement and maintain regular running behavior. This seems to be particularly the case for regular runners displaying positive IED (i.e., explicit >implicit) (42). Thus, positive affect might develop over time as individuals learn to like running regularly (16) while incorporating cognitive self-regulatory resources. In contrast, irregular runners are clearly represented in all four clusters. This demonstrates that implicit-explicit affective patterns can greatly vary for individuals who find it difficult to run regularly. While positive affect is important for regular running behavior, it is no guarantee for a successful implementation. Complementary to this, negative affect is not conducive to run regularly. However, it is also not a strict exclusion criterion to engage at least in irregular running. The omnipresence of irregular runners across all four clusters can be understood as a range of individual circumstances in terms of affective, intentional and motivational processes when it comes to irregular running behavior. When irregular runners are interpreted in terms of the IBG the results of the present study highlight the importance of behavior change strategies that consider several key factors that may very well vary among individuals who struggle to run regularly. Therefore, running interventions should follow an individualized approach that takes into account the interaction between implicit and explicit affect in addition to established cognitive self-regulation processes (6). Each cluster is discussed in terms of its prevalent IEI pattern and how this relates to motivational and intentional processes as well as running behavior.

### 4.1 “Positive non-discrepant” cluster 1

Participants assigned to the “positive non-discrepant” cluster 1 appear to like running without an affective conflict on the implicit-explicit level. This IEI pattern is theorized to be beneficial for goal achievement (i.e., translate intentions into action) since self-regulatory resources are not required to dissolve discrepant affective associations towards the target behavior (29, 47). Most participants in the “positive non-discrepant” cluster 1 were regular or irregular runners with regular running experience. Thus, past running experience might have supported or at least not hindered the formation of positive non-discrepant implicit and explicit associations towards running. Positive non-discrepant IEI accompanied by rather positive self-concordance towards running might favor initiation and maintenance of regular running behavior. In accordance, empirical work in the field of exercise psychology found higher values of IEC (38, 39) and small IED (40, 41) to coincide with more frequent and consistent exercise behavior. Interestingly, introjected motivation was moderate indicating involvement of guilty feelings in these participants’ motivation to run regularly (71). Feelings of guilt are considered to range on the negative affective dimension (82). Although the affective associations in the “positive non-discrepant” cluster 1 do not reflect this, high intention strength and effort readiness may indicate that regular running is not solely motivated by positive affect.

### 4.2 “Positive discrepant” cluster 2

In the “positive discrepant” cluster 2 IEI values indicate conflicting affective associations towards running. Here the observed direction of IED implies that participants explicitly state that they like running while holding rather negative associations on the implicit level. Young women assigned to “positive discrepant” cluster 2 — mainly regular and irregular runners with past regular running experience — might rather not enjoy running *per se*, assuming that implicit associations reflect the actual running experience (53). Nevertheless, they explicitly associate running with a positive feeling. Similar to the “positive non-discrepant” cluster 1, high scores of intrinsic, identified and introjected motivation were reported, while extrinsic motivation was rather irrelevant. Hence, the IEI pattern in the “positive discrepant” cluster 2 might in part reflect conflicting motivational aspects. Research demonstrated that large IED is linked to inflated exercise goals (39). Nine participants in the “positive discrepant” cluster 2 considered to start running regularly in the future. Thus, they currently do not meet their ideal running goals. Regular and irregular runners with large positive IED might realize their running behavior at the expense of self-control resources (29), since participants assigned to the “positive discrepant” cluster 2 indicated the highest values of intention strength and effort readiness of all four clusters. This is consistent with the results of a study where positive IED coincided with higher levels of PA when inhibitory control was high (42). Interventions aiming to make running more enjoyable may be beneficial for individuals with large IED, potentially leading to more positive implicit affective associations and more importantly to long-term running behavior.

### 4.3 “Negative discrepant” cluster 3

The IEI pattern of the “negative discrepant” cluster 3 suggests that participants explicitly have negative associations towards running, while holding rather positive implicit associations. From the theoretical perspective of a default-interventionist model (29, 44) this pattern would imply that getting presented with a target-related stimulus (i.e., running) triggers first a positive implicit affective association which is then followed and discarded by a reflective process resulting in the explicit report of a negative affective association. Most participants assigned to the “negative discrepant” cluster 3 have not been running recently and therefore, could not have formed any negative implicit affective association towards running based on running experience. Raw scores of implicit affective associations are mostly neutral (*M* = 0.07; *SD* = 0.17) and therefore could be interpreted as less negative than explicit affective associations instead of actual positive affective associations towards running. Also, intentional and motivational variables indicate that future engagement in regular running is rather unlikely (see Table 6). Running may be rather irrelevant to these participants on the implicit level, while being rejected on the explicit affective level to match their non-existent running behavior (i.e., “I do not run, because I do not like it.”). The irregular runners in the “negative discrepant” cluster 3 might not necessarily experience negative affect during running but do not seem to associate it positively on the explicit level. Since identified and introjected motivation scores were highest within this cluster — albeit only moderately in absolute terms — unrealistic running goals and feelings of guilt might drive the occasional and irregular running behavior.

### 4.4 “negative non-discrepant” cluster 4

From a dual-process perspective the negative non-discrepant IEI pattern found in cluster 4 is due to an implicitly activated negative affective association towards running formed by unpleasant past experiences and matched by congruent negative explicit affective associations (44, 46). It is unlikely that repeated negative affective experiences during running have formed this negative IEI pattern (i.e., negative IEC and small IED), since “negative non-discrepant” cluster 4 exclusively contains non-runners and irregular runners who have mostly no experience with regular running. Hence, the negative non-discrepant affective associations towards running must be based on other negative experiences. The IEI pattern of affective associations found in the “negative non-discrepant” cluster 4 is in line with research that links negative affect to exercise avoidance and inconsistent exercise behavior (29, 83–85). Possible reasons for these negative experiences with exercise that coincide with inactivity might be pain and breathlessness (83) which are well-known to be prevalent in running as well (86–88). Incisive interpersonal incidents like victimization during physical education at school might have contributed to negative affect and avoidance of running as well (89). In accordance, low values of intentional and motivational variables (see Table 6) indicate that these participants are not eager to engage in any future running activities.

### 4.5 Strengths & limitations

First, we would like to emphasize a few strengths of the present study. Fuzzy clustering offers an opportunity to explore and illustrate interacting implicit and explicit processes in a comprehensive manner taking their convoluted nature into account. The broad perspective on running behavior considering past, present and future aspects allowed a differentiated view on the complex relationship between affect and behavior. Further, targeting a specific domain of exercise like running, in contrast to general physical activity, probably yields more accurate results since individuals tend to have ambivalent affective associations towards different exercise domains (10, 11, 48, 49). Also, the online data collection might have prevented unwanted influence on measurement of implicit associations by exercise context like sport research facilities (38, 44). On the other hand, some limitations of this work need to be addressed. While holding advantages, collecting data online usually comes at the price of reduced standardization and controllability of measurement. In case of the ST-IAT using different presentation devices additional sources of measurement error (e.g., varying stimulus size, varying accuracy in RT collection) are most likely present in the data. Running behavior was quantified by self-report only, which is subject to influences of social desirability, recall bias and are inferior to objective measures in terms of validity (90–95). Aside from running, most participants in this study reported to be generally physically active (96.6% pursuit some sort of exercise). Investigation of inactive women might further validate the importance of implicit and explicit affective associations for strategies to promote physical activity (40). Especially unexperienced running intenders might profit from interventions that consider implicit and explicit affective processes (5). When considering beginners in running, it may be worthwhile to explore self-paced running that prioritises creating an enjoyable experience over emphasising performance. Also, implementing the concept of subjective vitality (i.e., an activated positive affective state of feeling energized) as target outcome for running programs might facilitate the desired convergence of action initiation and positive affect (96, 97). Another aspect that demands further investigation is the measurement of implicit affective associations towards running. In this study, implicit associations were quantified based on behavioral data derived from differences in RT of opposing affective association conditions in a sorting task (ST-IAT). This approach is commonly used despite well-known limitations in both reliability and validity (98–100). Event-related potential (ERP) components collected from concurrent electroencephalography (EEG) as indices of both affective and implicit processing yield promising results (101–103). Assessing implicit affective associations towards running derived from ERP components regarding implicit-explicit interaction and running behavior might further advance this endeavor.

## 5 Conclusion

The results of the present study demonstrate that running behavior can vary within IEI patterns. For instance, among regular runners some hold discrepant implicit and explicit associations while others do not. Similar relations were found for irregular runners as well as non-runners. These findings support the point of view that the relationship between affect and exercise behavior is more complex than the oversimplified “exercise makes people feel better” statement (83). Overlap between clusters enabled by the fuzzy clustering approach indicate that straightforward assignments of IEI patterns might underestimate the interactive complexity of affective associations. Thus, classifying individuals based on their affective IEI patterns is not recommended. Future research should address the question whether IEI patterns truly hold the potential to inform individualized (running) interventions that target affective processes. Conditions under which individuals with running intentions are willing to engage in interventions that target affective processes based on their individual implicit and explicit affective associations should be investigated as well. Finally, the present findings once again confirm that considering implicit besides explicit affective processes in exercise psychological research is warranted.

## Data Availability

The datasets presented in this article are not readily available because of sharing agreements in the funded project. Requests to access the datasets should be directed to tim.burberg@plus.ac.at.
