# Supplementary material for: Affective associations towards running: fuzzy patterns of implicit-explicit interaction in young female runners and non-runners
Source: Front Sports Act Living. 2024 Jan 31;6:1210546. doi: 10.3389/fspor.2024.1210546 (PMC10864672; doi:10.3389/fspor.2024.1210546)
Supplement: Supplementary file 1 [file Datasheet1.docx]

Supplementary Material 1

Affective associations towards running: Fuzzy patterns of implicit-explicit interaction in young female runners and non-runners

Tim Burberg*, Sabine Würth, Günter Amesberger and Thomas Finkenzeller

*** Correspondence:**

Tim Burberg
tim.burberg@plus.ac.at

# Stimulus Material of the ST-IAT

Emojis have already been used as affective stimuli in implicit measures like the ST-IAT (e.g., 1). In the present study smileys and frownies from the operating system iOS were selected based on subjective ratings from the Lisbon Emoji and Emoticon Database (LEED) (2). Rodrigues and colleagues (2) had n = 505 individuals rate a randomly assigned subset of commonly used emojis from several operating systems on a bipolar seven-point scale in seven evaluative dimensions (i.e., aesthetic appeal, familiarity, visual complexity, clarity, valence, arousal and meaningfulness). We deemed the evaluative dimensions clarity, valence and arousal relevant for emojis to adequately represent affective ST-IAT categories (3). According to Rodrigues and colleagues (2) clarity was defined as the degree to which an emoji clearly conveys an emotion/meaning ranging from 1 = “totally ambiguous” to 7 = “totally clear”. Valence was instructed to reflect the extent of how positive/pleasant or negative/unpleasant an emoji was perceived with 1 = “very negative” and 7 = “very positive”. The dimension of arousal addressed to what extent an emoji evoked an exciting or calm feeling (1 = “very passive/calm”, 7 = “very arousing/exciting”). Rodrigues and colleagues (2) categorized all emojis as “low”, “moderate” or “high” based on whether the confidence interval included the scale midpoint of 4 or not. In general, emojis rated “high” on the evaluative dimensions clarity and arousal were considered eligible. Ultimately, emojis categorized as “high” and “low” on the valence dimension were labeled as smileys and frownies, respectively. A pre-selection of eight smileys and frownies each was internally discussed and finally four simleys and frownies each were implemented as affective stimuli in the ST-IAT by consensus (see Table 1 and 2).

**Supplementary Table 1.1** *Illustration and description of frownies representing the negative affective category in the ST-IAT by stimulus size and LEED rating.*

| Unicode | Stimulus | Stimulus size [px] | |  | Stimulus evaluation *M* (*SD*) | | |
| --- | --- | --- | --- | --- | --- | --- | --- |
|  |  | height | width |  | clarity | valence | arousal |
| U+2639 | 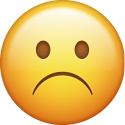 | 125 | 125 |  | 6.33 (0.93) | 2.02 (1.02) | 4.71 (1.57) |
| U+1F620 | 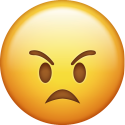 | 125 | 125 |  | 6.00 (1.26) | 2.05 (1.63) | 6.25 (0.93) |
| U+1F62B | 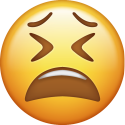 | 125 | 125 |  | 5.15 (1.53) | 2.00 (1.30) | 5.66 (1.48) |
| U+1F629 | 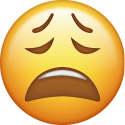 | 125 | 125 |  | 5.14 (1.46) | 2.29 (1.20) | 5.00 (1.65) |

**Supplementary Table 1.2** *Illustration and description of smileys representing the positive affective category in the ST-IAT by stimulus size and LEED rating.*

| Unicode | Stimulus | Stimulus size [px] | |  | Stimulus evaluation *M* (*SD*) | | |
| --- | --- | --- | --- | --- | --- | --- | --- |
|  |  | height | width |  | clarity | valence | arousal |
| U+1F603 | 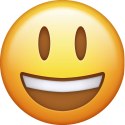 | 125 | 125 |  | 6.68 (0.69) | 6.71 (0.64) | 6.10 (1.36) |
| U+1F60A | 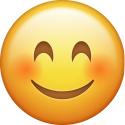 | 125 | 125 |  | 6.20 (1.15) | 6.29 (0.90) | 4.68 (1.78) |
| U+1F600 | 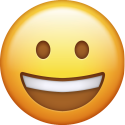 | 125 | 125 |  | 6.20 (1.36) | 6.12 (1.44) | 5.29 (1.75) |
| U+1F604 | 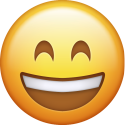 | 125 | 125 |  | 6.49 (0.97) | 6.60 (0.72) | 6.16 (1.04) |

**Supplementary Table 1.3** *Illustration and description of female runner silhouettes representing the target category “Running” in the ST-IAT by stimulus size.*

| Stimulus name | Stimulus | Stimulus size [px] | |
| --- | --- | --- | --- |
|  |  | height | width |
| Runner01 | 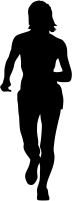 | 201 | 72 |
| Runner02 | 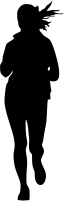 | 201 | 64 |
| Runner03 | 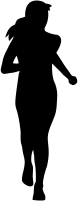 | 201 | 78 |
| Runner04 | 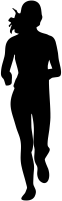 | 201 | 62 |

**References**

1. Brand R, Heck P, Ziegler M. Illegal performance enhancing drugs and doping in sport: a picture-based brief implicit association test for measuring athletes’ attitudes. Substance abuse treatment, prevention, and policy. 2014 Dec;9(1):1-1.

2. Rodrigues D, Prada M, Gaspar R, Garrido MV, Lopes D. Lisbon Emoji and Emoticon Database (LEED): Norms for emoji and emoticons in seven evaluative dimensions. Behavior research methods. 2018 Feb;50:392-405.

3. Nosek BA, Greenwald AG, Banaji MR. Understanding and using the Implicit Association Test: II. Method variables and construct validity. Personality and Social Psychology Bulletin. 2005 Feb;31(2):166-80.

**
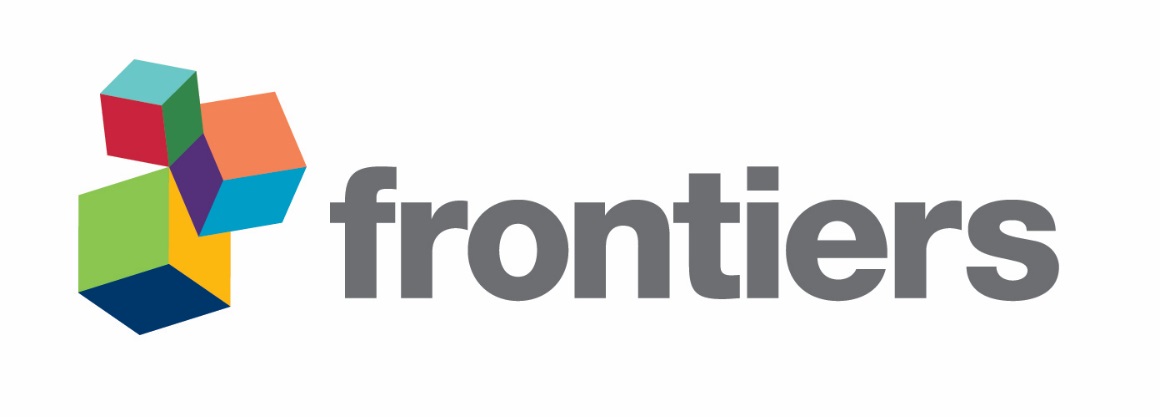
**
