# Supplementary material for: Affective associations towards running: fuzzy patterns of implicit-explicit interaction in young female runners and non-runners
Source: Front Sports Act Living. 2024 Jan 31;6:1210546. doi: 10.3389/fspor.2024.1210546 (PMC10864672; doi:10.3389/fspor.2024.1210546)
Supplement: Supplementary file 2 [file Datasheet2.docx]

Supplementary Material 2

Affective associations towards running: Fuzzy patterns of implicit-explicit interaction in young female runners and non-runners

Tim Burberg*, Sabine Würth, Günter Amesberger and Thomas Finkenzeller

*** Correspondence:**

Tim Burberg
tim.burberg@plus.ac.at

# German versions of self-report measures

**Supplementary Table 2.1** *German instructions and items used to assess handedness based on the Edinburgh Handedness Inventory by Oldfield (1).*

| „Wähle nachfolgend bitte jene Kästchen aus, welche am besten beschreiben, welche Hand du für die jeweilige Aktivität verwendest:“ | | | | | |
| --- | --- | --- | --- | --- | --- |
|  | Immer  links  (-2) | Gewöhnlich links  (-1) | Keine Präferenz  (0) | Gewöhnlich rechts  (1) | Immer rechts  (2) |
| Schreiben |  |  |  |  |  |
| Werfen |  |  |  |  |  |
| Schneiden mit der Schere |  |  |  |  |  |
| Zähneputzen |  |  |  |  |  |
| Schneiden mit dem Messer |  |  |  |  |  |
| Löffel benutzen |  |  |  |  |  |
| Streichholz anzünden |  |  |  |  |  |
| Computermaus benutzen |  |  |  |  |  |

**Supplementary Table 2.2** *German instruction and illustration of the “Feeling Thermometer” scale to measure explicit affective associations towards running.*

| „Wie fühlst du dich beim Laufen? Bitte bewerte dein Gefühl anhand der abgebildeten Skala.“ | | | | | | | | | | | | | | |
| --- | --- | --- | --- | --- | --- | --- | --- | --- | --- | --- | --- | --- | --- | --- |
| Skalenbeschriftung | | | | | | | | | | | | | | |
| unangenehm/ negativ | | |  | | | neutral | | |  | | | angenehm/ positiv | | |
| 0 | 1 | 2 | | 3 | 4 | | 5 | 6 | | 7 | 8 | | 9 | 10 |

**Supplementary Table 2.3** *German instruction and illustration of items used to quantify past regular running experience, current running behavior and main exercise.*

| „Nun bitten wir dich um einige Angaben zu deiner sportlichen Biographie:“ |
| --- |
| 1. Erfahrungen mit der Sportart Laufen (bitte nur eine der folgenden Aussagen anklicken): |
| - Ich bin nie regelmäßig gelaufen und will auch nicht damit beginnen. - Ich bin früher regelmäßig gelaufen, will aber aktuell nicht wieder damit beginnen. - Ich bin nie regelmäßig gelaufen, überlege aber damit anzufangen. - Ich bin früher regelmäßig gelaufen und überlege, wieder damit anzufangen. - Ich laufe regelmäßig. |
| 2. Aktuelles Laufverhalten (bitte nur eine der folgenden Aussagen anklicken): |
| - Ich laufe nicht. - Ich laufe aktuell unregelmäßig. - Ich laufe regelmäßig seit maximal einem Monat* - Ich laufe regelmäßig seit maximal drei Monaten* - Ich laufe regelmäßig seit maximal sechs Monaten - Ich laufe regelmäßig seit maximal einem Jahr - Ich laufe regelmäßig seit mehr als einem Jahr |
| 3. Sportart, die du hauptsächlich betreibst (bitte nur eine nennen): |

* Participants indicating to be currently running regularly for one or three months were not included in the analysis.

**Supplementary Table 2.4** *German instruction and modified items used to measure running-related intention strength and effort readiness according to Seelig & Fuchs (2).*

| 1. „Wie stark ist deine Absicht, in den nächsten Wochen und Monaten regelmäßig laufen zu gehen?“ | | | | | | | | | | | |
| --- | --- | --- | --- | --- | --- | --- | --- | --- | --- | --- | --- |
| Gar nicht stark | | | | |  | | | | Ganz stark | | |
| 1 | 2 | 3 | 4 | 5 | | 6 | 7 | 8 | | 9 | 10 |
| 2. „Wie viel Anstrengung wärst du bereit aufzubringen, um in den nächsten Wochen und Monaten regelmäßig laufen gehen zu können?“ | | | | | | | | | | | |
| Gar keine | | | |  | |  | Sehr große | | | | |
| 1 | 2 | 3 | 4 | 5 | | 6 | 7 | 8 | | 9 | 10 |

**Supplementary Table 2.5** *German instruction and modified items used to measure running-related intention strength and effort readiness according to Seelig & Fuchs (2).*

| „Im Folgenden findest Du einige Aussagen, die sich auf das regelmäßige Laufen beziehen. Wie treffen diese Aussagen auf dich zu?“ | | | | | | | | |
| --- | --- | --- | --- | --- | --- | --- | --- | --- |
| Item | | Trifft gar nicht zu | | | | Trifft genau zu | | |
| Nr. | Formulierung | 1 | 2 | 3 | 4 | | 5 | 6 |
| 1 | Laufen gehen gehört einfach zu meinem Leben dazu. |  |  |  |  | |  |  |
| 2 | Laufen macht mir einfach Spaß. |  |  |  |  | |  |  |
| 3 | Beim Laufen mache ich Erfahrungen, die ich nicht missen möchte. |  |  |  |  | |  |  |
| 4 | Ich habe gute Gründe Laufen zu gehen. |  |  |  |  | |  |  |
| 5 | Laufen ist gut für mich. |  |  |  |  | |  |  |
| 6 | Die positiven Folgen sind einfach die Mühe wert. |  |  |  |  | |  |  |
| 7 | Ich hätte sonst ein schlechtes Gewissen, wenn ich nicht laufen gehen würde. |  |  |  |  | |  |  |
| 8 | Ich denke, dass man sich manchmal zu etwas zwingen muss. |  |  |  |  | |  |  |
| 9 | Ich müsste mir sonst Vorwürfe machen, wenn ich nicht laufen gehen würde. |  |  |  |  | |  |  |
| 10 | Personen, die mir wichtig sind, drängen mich zum Laufen. |  |  |  |  | |  |  |
| 11 | Ich bekomme sonst mit anderen Personen Schwierigkeiten, wenn ich nicht laufen gehen würde. |  |  |  |  | |  |  |
| 12 | Andere sagen, ich soll laufen gehen. |  |  |  |  | |  |  |

**References**

1. Oldfield RC. The assessment and analysis of handedness: the Edinburgh inventory. Neuropsychologia. 1971 Mar 1;9(1):97-113.

2. Seelig, H., & Fuchs, R. (2006). Messung der sport-und bewegungsbezogenen Selbstkonkordanz. *Zeitschrift für Sportpsychologie*, *13*(4), 121-139.

**
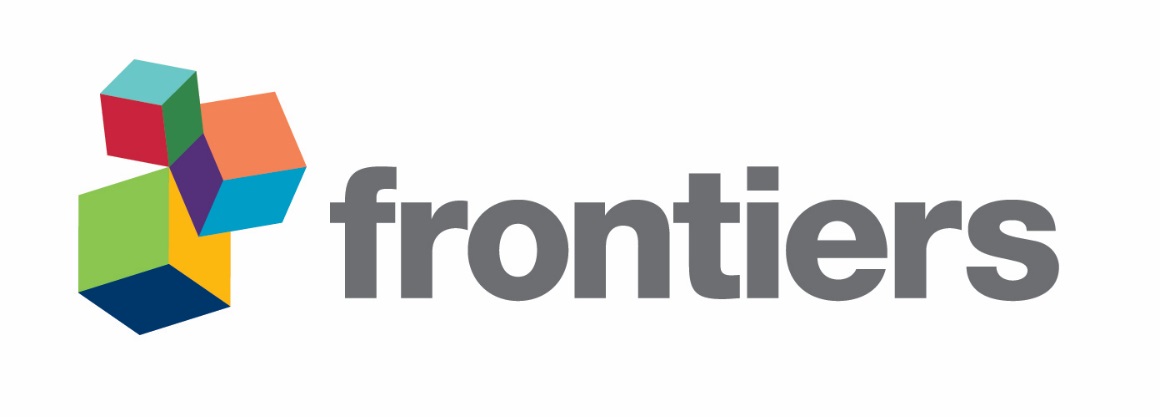
**
