# Supplementary material for: Affective associations towards running: fuzzy patterns of implicit-explicit interaction in young female runners and non-runners
Source: Front Sports Act Living. 2024 Jan 31;6:1210546. doi: 10.3389/fspor.2024.1210546 (PMC10864672; doi:10.3389/fspor.2024.1210546)
Supplement: Supplementary file 3 [file Datasheet3.docx]

Supplementary Material 3

Affective associations towards running: Fuzzy patterns of implicit-explicit interaction in young female runners and non-runners

Tim Burberg*, Sabine Würth, Günter Amesberger and Thomas Finkenzeller

*** Correspondence:**

Tim Burberg
tim.burberg@plus.ac.at

# Cluster analysis

**Supplementary Figure 3.1** *Dendrogram derived from Ward’s hierarchical clustering to determine number of clusters.*

**
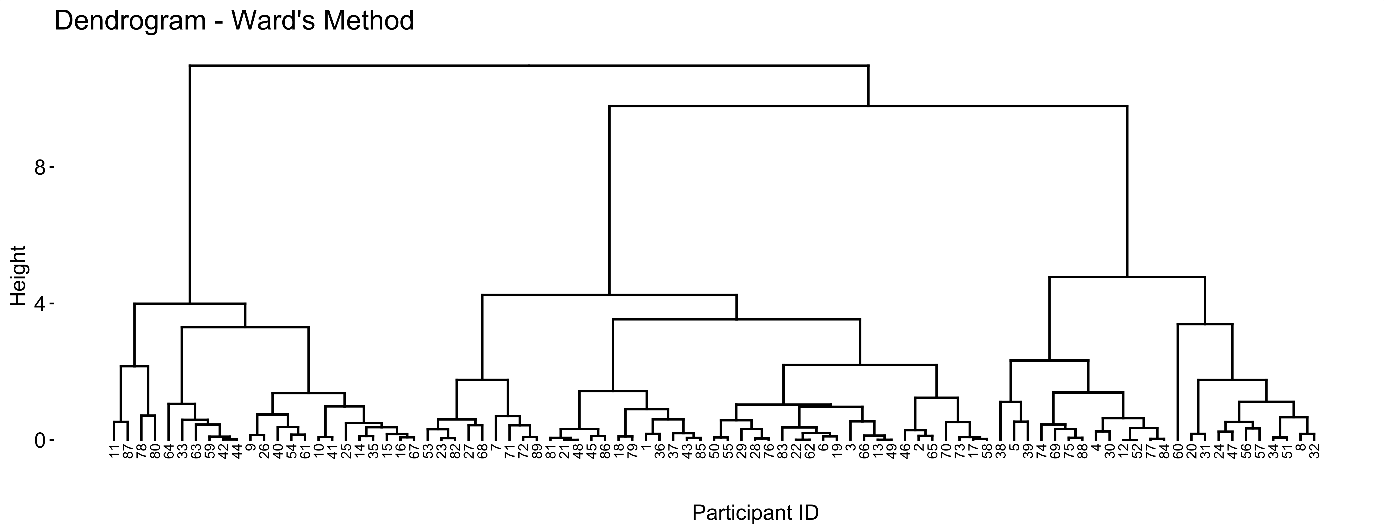
**

**Supplementary Figure 3.2** *Illustration of implicit-explicit interaction grouped by crisp k-means cluster solution (k = 3).*

**
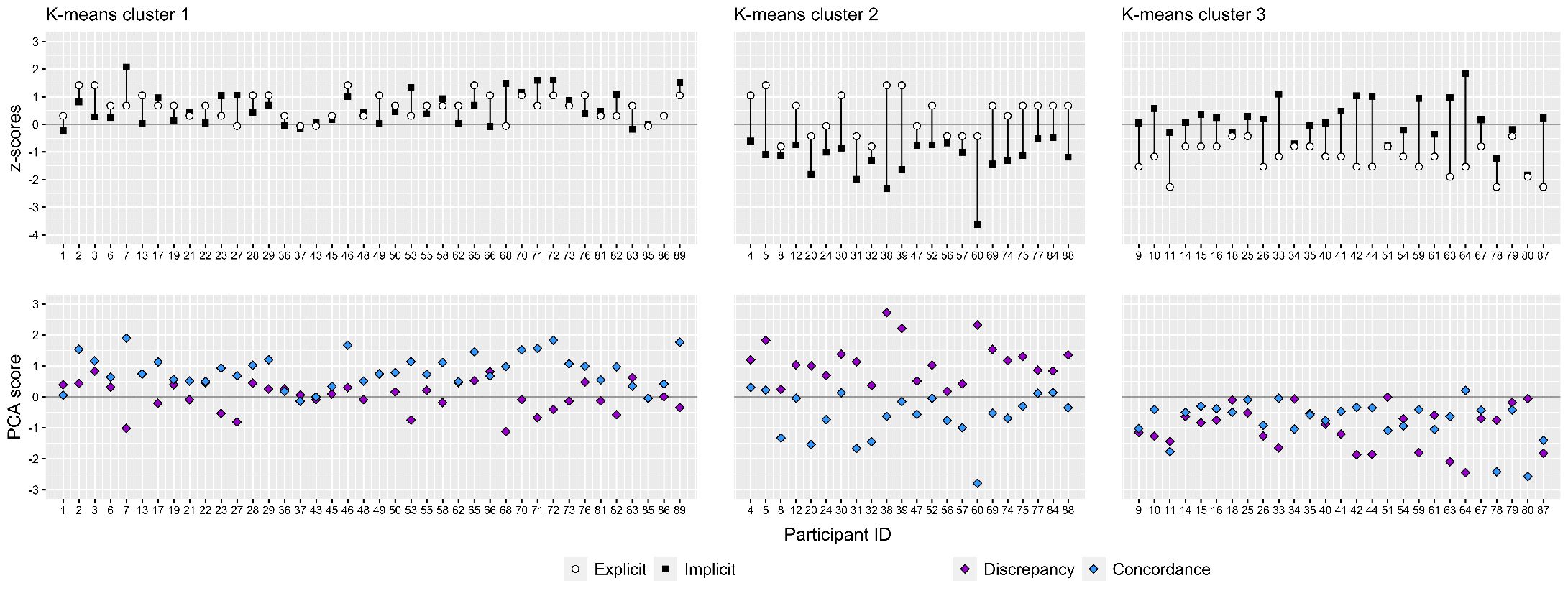
**

**Supplementary Figure 3.3** *Illustration of implicit-explicit interaction grouped by crisp k-means cluster solution (k = 4).*

**
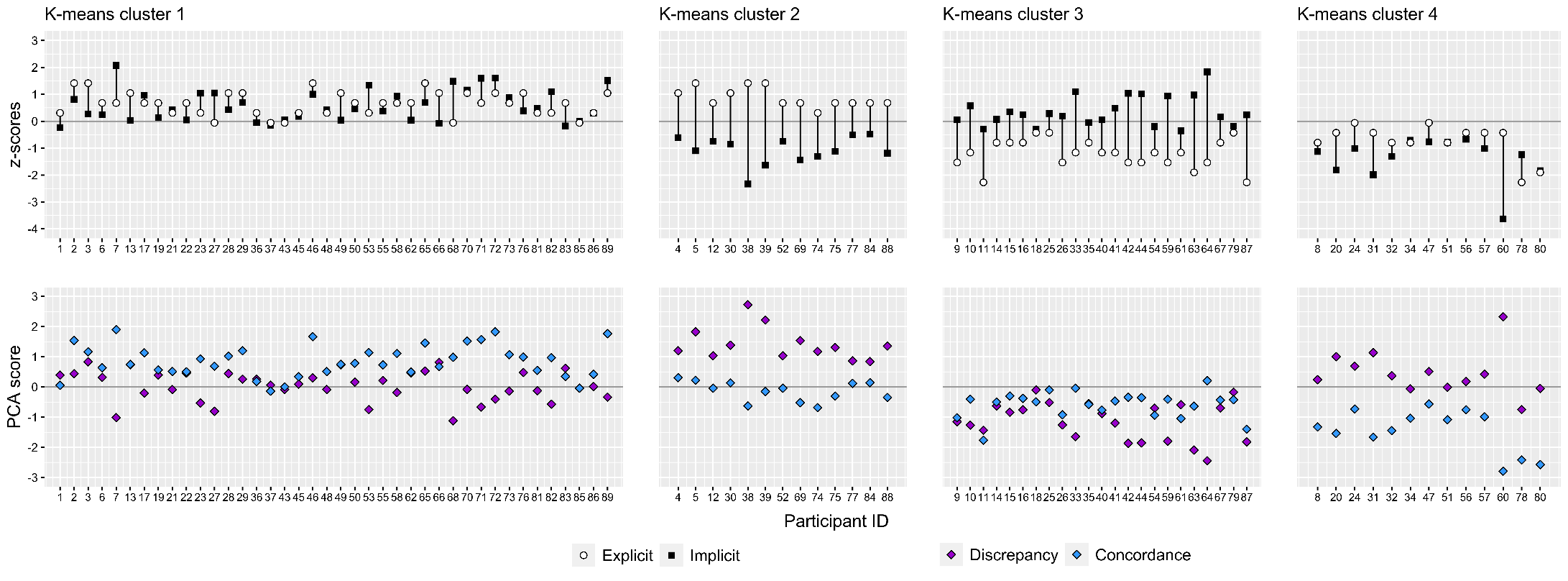
**

**Supplementary Table 3.1** *Descriptive and distributional information on intrinsic, identified and introjected motivation for fuzzy clusters.*

|  | Nr. | *M* | *SD* | *Mdn* | Range | Skew | Kurtosis |  | Shapiro-wilk test | |  | Levene’s test | |
| --- | --- | --- | --- | --- | --- | --- | --- | --- | --- | --- | --- | --- | --- |
|  |  |  |  |  |  |  |  |  | *W* | *p* |  | *F*(3, 85) | *p* |
| **Intrinsic motivation** |  |  |  |  |  |  |  |  |  |  |  | 1.17 | .327 |
| Positive non-discrepant | 1 | 4.20 | 1.25 | 4.50 | 1.33 – 6.00 | -0.82 | -0.27 |  | 0.90 | **.004** |  |  |  |
| Positive discrepant | 2 | 4.52 | 1.18 | 4.83 | 2.67 – 6.00 | -0.35 | -1.38 |  | 0.90 | .069 |  |  |  |
| Negative discrepant | 3 | 1.68 | 0.88 | 1.33 | 1.00 - 4.00 | 1.27 | 0.50 |  | 0.78 | **< .001** |  |  |  |
| Negative non-discrepant | 4 | 2.18 | 0.92 | 2.00 | 1.00 - 4.00 | 0.43 | -0.98 |  | 0.94 | .320 |  |  |  |
| **Identified motivation** |  |  |  |  |  |  |  |  |  |  |  | 4.67 | **.005** |
| Positive non-discrepant | 1 | 5.14 | 0.78 | 5.17 | 3.00 – 6.00 | -0.74 | -0.14 |  | 0.90 | **.004** |  |  |  |
| Positive discrepant | 2 | 5.48 | 0.53 | 5.67 | 4.67 – 6.00 | -0.41 | -1.53 |  | 0.83 | **.008** |  |  |  |
| Negative discrepant | 3 | 2.93 | 1.45 | 2.83 | 1.00 – 6.00 | 0.40 | -0.83 |  | 0.95 | .424 |  |  |  |
| Negative non-discrepant | 4 | 3.59 | 1.04 | 4.00 | 1.67 – 5.00 | -0.46 | -1.33 |  | 0.91 | .093 |  |  |  |
| **Introjected motivation** |  |  |  |  |  |  |  |  |  |  |  | 1.08 | .363 |
| Positive non-discrepant | 1 | 3.41 | 0.89 | 3.33 | 1.67 – 5.33 | 0.06 | -0.91 |  | 0.97 | .421 |  |  |  |
| Positive discrepant | 2 | 4.04 | 1.18 | 4.00 | 2.00 – 5.67 | 0.01 | -1.36 |  | 0.94 | .361 |  |  |  |
| Negative discrepant | 3 | 2.35 | 1.32 | 2.17 | 1.00 – 5.67 | 0.86 | -0.18 |  | 0.89 | .026 |  |  |  |
| Negative non-discrepant | 4 | 2.59 | 0.98 | 2.67 | 1.00 – 4.33 | -0.07 | -1.07 |  | 0.96 | .635 |  |  |  |

*p* < .050 in bold font.

**Supplementary Table 3.2** *Descriptive and distributional information on extrinsic motivation, self-concordance and intention strength for fuzzy clusters.*

|  | Nr. | *M* | *SD* | *Mdn* | Range | Skew | Kurtosis |  | Shapiro-wilk test | |  | Levene’s test | |
| --- | --- | --- | --- | --- | --- | --- | --- | --- | --- | --- | --- | --- | --- |
|  |  |  |  |  |  |  |  |  | *W* | *p* |  | *F*(3, 85) | *p* |
| **Extrinsic motivation** |  |  |  |  |  |  |  |  |  |  |  | 0.93 | .432 |
| Positive non-discrepant | 1 | 1.52 | 0.81 | 1.00 | 1.00 – 4.00 | 1.52 | 1.32 |  | 0.70 | **< .001** |  |  |  |
| Positive discrepant | 2 | 1.19 | 0.52 | 1.00 | 1.00 – 3.00 | 2.72 | 6.58 |  | 0.43 | **< .001** |  |  |  |
| Negative discrepant | 3 | 1.57 | 1.01 | 1.00 | 1.00 – 4.33 | 1.77 | 1.77 |  | 0.62 | **< .001** |  |  |  |
| Negative non-discrepant | 4 | 1.33 | 0.68 | 1.00 | 1.00 – 3.67 | 2.40 | 5.40 |  | 0.57 | **< .001** |  |  |  |
| **Self-concordance** |  |  |  |  |  |  |  |  |  |  |  | 1.82 | .150 |
| Positive non-discrepant | 1 | 4.42 | 1.69 | 4.50 | -0.33 – 8.00 | -0.45 | 0.57 |  | 0.97 | .379 |  |  |  |
| Positive discrepant | 2 | 4.77 | 1.59 | 4.50 | 2.33 - 7.33 | 0.09 | -1.06 |  | 0.93 | .237 |  |  |  |
| Negative discrepant | 3 | 0.70 | 1.12 | 0.67 | -1.33 – 3.67 | 0.60 | 0.50 |  | 0.95 | .436 |  |  |  |
| Negative non-discrepant | 4 | 1.84 | 2.12 | 1.33 | -2.00 – 6.33 | 0.21 | -0.52 |  | 0.98 | .974 |  |  |  |
| **Intention strength** |  |  |  |  |  |  |  |  |  |  |  | 0.15 | .931 |
| Positive non-discrepant | 1 | 7.56 | 2.51 | 8.00 | 1.00 – 10.00 | -1.06 | 0.06 |  | 0.84 | **< .001** |  |  |  |
| Positive discrepant | 2 | 8.00 | 2.16 | 8.50 | 2.00 – 10.00 | -1.19 | 1.06 |  | 0.84 | **.010** |  |  |  |
| Negative discrepant | 3 | 3.10 | 2.73 | 2.00 | 1.00 – 10.00 | 1.21 | 0.10 |  | 0.76 | **< .001** |  |  |  |
| Negative non-discrepant | 4 | 3.41 | 1.87 | 3.00 | 1.00 – 7.00 | 0.24 | -1.08 |  | 0.93 | .180 |  |  |  |

*p* < .050 in bold font.

**Supplementary Table 3.3** *Descriptive and distributional information on effort readiness for fuzzy clusters.*

|  | Nr. | *M* | *SD* | *Mdn* | Range | Skew | Kurtosis |  | Shapiro-wilk test | |  | Levene’s test | |
| --- | --- | --- | --- | --- | --- | --- | --- | --- | --- | --- | --- | --- | --- |
|  |  |  |  |  |  |  |  |  | *W* | *p* |  | *F*(3, 85) | *p* |
| **Effort readiness** |  |  |  |  |  |  |  |  |  |  |  | 2.40 | .073 |
| Positive non-discrepant | 1 | 7.42 | 1.84 | 8.00 | 3.00 – 10.00 | -0.47 | -0.46 |  | 0.92 | **.015** |  |  |  |
| Positive discrepant | 2 | 7.81 | 1.68 | 8.00 | 4.00 – 10.00 | -0.43 | -0.59 |  | 0.93 | .281 |  |  |  |
| Negative discrepant | 3 | 4.25 | 2.67 | 4.00 | 1.00 – 10.00 | 0.33 | -1.12 |  | 0.91 | .066 |  |  |  |
| Negative non-discrepant | 4 | 4.35 | 4.35 | 5.00 | 1.00 – 7.00 | -0.13 | -1.37 |  | 0.93 | .231 |  |  |  |

*p* < .050 in bold font.
